# Multicentric short term and safety study of ineffective esophageal motility patients treated with RefluxStop device

**DOI:** 10.1038/s41598-024-65751-5

**Published:** 2024-07-04

**Authors:** J. Feka, M. Saad, N. Boyle, M. Paireder, I. Kristo, E. Rieder, R. Asari, S. F. Schoppmann

**Affiliations:** 1 Department of Surgery, Division of General Surgery, Comprehensive Cancer Center, Upper-GI Unit, Medical University of Vienna, Spitalgasse 23, Waehringer Guertel 18-20, 1090 Vienna, Austria; 2 Department of Surgery, King Edward VII’s Hospital, London, UK

**Keywords:** GERD, Dysphagia, RefluxStop, His angle reconstruction, Motility disorders, Gastro-oesophageal reflux disease, Outcomes research, Medical research, Signs and symptoms

## Abstract

Gastroesophageal reflux disease (GERD) presents a general health problem with a variety of symptoms and an impairment of life quality. Conservative therapies do not offer sufficient symptom relief in up to 30% of patients. Patients who suffer from ineffective esophageal motility (IEM) and also GERD may exhibit symptoms ranging from mild to severe. In cases where surgical intervention becomes necessary for this diverse group of patients, it is important to consider the potential occurrence of postoperative dysphagia. RefluxStop is a new alternative anti-reflux surgery potentially reducing postoperative dysphagia rates. In this bicentric tertiary hospital observational study consecutive patients diagnosed with PPI refractory GERD and IEM that received RefluxStop implantation were included. A first safety and efficacy evaluation including clinical examination and GERD-HRQL questionnaire was conducted. 40 patients (25 male and 15 female) were included. 31 patients (77.5%) were on PPI at time of surgery, with mean acid exposure time of 8.14% ± 2.53. The median hospital stay was 3 days. Postoperative QoL improved significantly measured by GERD HRQL total score from 32.83 ± 5.08 to 6.6 ± 3.71 (p < 0.001). A 84% reduction of PPI usage (p < 0.001) was noted. 36 patients (90%) showed gone or improved symptoms and were satisfied at first follow-up. Two severe adverse events need mentioning: one postoperative slipping of the RefluxStop with need of immediate revisional operation on the first postoperative day (Clavien–Dindo Score 3b) and one device migration with no necessary further intervention. RefluxStop device implantation is safe and efficient in the short term follow up in patients with GERD and IEM. Further studies and longer follow-up are necessary to prove long-lasting positive effects.

## Introduction

Gastroesophageal reflux disease (GERD) is one of the most common diseases worldwide with prevalence rates up to 33%^1^. Around 1 billion people throughout all continents are currently suffering from GERD^2^.

Up to 30% of these patients suffer from PPI therapy refractory GERD, meaning that no symptom relief is achieved by lifestyle modifications and medication^3^. If conservative therapy has been exhausted, the next step is considered to be anti-reflux surgery^4^. Much has been improved since the beginning of Nissen fundoplication over 60 years ago: nowadays, surgeons may choose between total and partial fundoplication, magnetic sphincter augmentation, and anti-reflux mucosectomy, just to name a few procedures^4^.

Patients with long time GERD are reported to have a higher likelihood to suffer from esophageal motility disorder, such as ineffective esophageal motility (IEM)^5^. This disorder is defined by the Chicago classification v4.0 as more than 70% ineffective swallows or at least 50% failed peristalsis with a normal integrated relaxation pressure (IRP)^6^. This patient group consisting of up to 36% of GERD patients is heterogenous in clinical presentation^7–10^. It is suggested, that IEM might subsequently lead to worse abnormal acid burden, heartburn, regurgitation and to a certain degree dysphagia^5,11^. The inconsistency in symptoms, especially in regard to dysphagia, makes this patient group quite difficult to treat adequately. Even though various studies could show similar adverse events with different surgical techniques, the problem of postoperative dysphagia is still under debate in IEM patients undergoing anti-reflux surgery^12–14^. A new anti-reflux surgical technique is intended to prevent dysphagia postoperatively: the implantation of the RefluxStop^™^, a silicon, single-use, sterile, non-active implantable device^15^. This new procedure targets two different but crucial points, which might be in charge of GERD development. Firstly, the misaligned angle of His and secondly, the movement of the lower esophageal sphincter (LES), whether temporarily or permanently, into the thorax^15^.

Despite the novelty of this procedure, the first experiences have been favorable: In a one-year follow-up after RefluxStop^™^ device implantation, Bjelović et al. were able to show a significant improvement in 24 h pH monitoring compared to preoperative findings, no dysphagia as well as overall patient satisfaction^15^.

Aim of this study was to evaluate the safety and short term efficacy of RefluxStop in patients with GERD and IEM.

## Methods

### Patient collective

Consecutive patients (n = 40) with GERD received a RefluxStop^™^ device implantation at the King Edward VII Hospital in London (n = 16) and at the General Hospital of the Medical University of Vienna (n = 24), respectively, in the period of May 2021 and February 2023. All patients had ineffective esophageal motility according to the Chicago classification v4.0 on high-resolution manometry. The analysis included clinicopathological factors, intraoperative complications, length of hospital stay, PPI usage and satisfaction score preoperatively, barium swallow postoperatively, and the evaluation of the GERD-health related quality of life (GERD-HRQL) questionnaire^16^. All patients received esophageal function testing prior surgery.

Inclusion criteria included indication for anti-reflux surgery: more than one year of GERD, history of PPI usage, IEM criteria according to Chicago classification v4.0 and GERD diagnosis via 24 h impedence pH testing. Exclusion criteria entail patients with any history of esophageal or gastric malignancies, any type of esophageal strictures or stenosis and patients unwilling to attend follow-up. Complete eligibility criteria found in Table 1.

**Table 1:** Eligibility criteria of patient cohort.

Inclusion criteria	Exclusion criteria
Age > 18 years	Any esophageal or gastric malignancy
Years of GERD > 1 year	Any esophageal stenosis or stricture
History of PPI Usage	Patients unwilling to attend follow-up
Ineffective esophageal motility	Patients allergic to any parts of RefluxStop^™^ device
GERD diagnosis via 24 h impedance pH testing	
Body mass index (BMI) < 35 m^2^/kg	

The primary endpoint of this analysis was short-term clinical outcome, defined by GERD symptom improvement, measured by subjective satisfaction and GERD-HRQL score. The secondary endpoint was to investigate postoperative side effects, especially with regard to dysphagia.

### Surgical information

This new procedure can be conducted for any GERD patient, who fits the normal criteria for any anti-reflux surgery^15^. The patient is fully anesthetized, placed in reverse Trendelenburg position with the lower extremities abducted, properly washed and draped, as for any kind of laparoscopic surgery^17^. After extensive and as high up preparation of the esophagus up into the mediastinum, and therefore achieving the return of the LES into the abdominal cavity, the cruroplasty is performed as usual, either with interrupted or continuous sutures. Next, the reconstruction of the His angle commences^17^. When a floppy fundus is achieved, it is then sutured onto the esophagus all the way up to the diaphragm with non-resorbable, interrupted sutures in two rows, while keeping the vagal nerves intact^17^. A minimum length of 4 cm and the removal of any fatty tissue at the gastroesophageal junction are advised in order to achieve maximum impact^17^. Subsequently, the implantation of the RefluxStop^™^ device is carried out: this is a single-use, sterile and non-active device and an assembly of five small silicon parts, which are tied together by a resorbable suture^17^. This is done intraoperatively at the back table. The device is introduced into the abdominal cavity via a specially made trocar on a deployment tool and placed on the anterior fundus wall. The invagination of the device is achieved with a few interrupted tobacco pouch sutures, until the device is not visible anymore^17^. Positioning the device as high above of the LES as possible is essential for positive outcome postoperatively^17^. The deployment tool is then removed and the operation is concluded as usual.

### Statistical analysis

Excel (version 16.69.1) was used for statistical analysis. All variables were presented as the mean values ± standard deviation or median and interquartile range or 95% confidence intervals (Cis). Statistical analysis was based on paired *t*-test, McNemar test and χ2 test, as appropriate. p values < 0.05 were considered significant. Graphing was performed with Excel.

All methods were carried out in accordance with relevant guidelines, regulations and ethical approval. Individual informed consent was not acquired, due to study design and national regulations. The study was approved by the ethics committee (EK 1438/2023) of the Medical University of Vienna and by the Medical Advisory Committee at the King Edward VII's hospital.

The datasets used and analyzed during the current study available from the corresponding author on reasonable request.

## RESULTS

### Patients

Forty patients received a RefluxStop^™^ device implantation at the King Edward VII Hospital in London (n = 16) and at the General Hospital of the Medical University of Vienna (n = 24) in the period of May 2021 and February 2023. 15 were female (37.5%), 25 male (62.5%). Mean age was 48.93 ± 4.59 years. Patients were suffering from GERD for a mean of 6.44 ± 1.57 years, and mean PPI intake was 3.5 ± 0.84 years. At time of surgery, 31 patients (77.5%) were taking PPI on a daily basis. Patients presented with a variety of leading symptoms, most commonly with heartburn, followed by regurgitation, thoracic or abdominal pain, laryngopharyngeal reflux and dysphagia. Four patients (10%) reported temporary dysphagia as leading symptom prior surgery. Reflux esophagitis in preoperative gastroscopy and in accordance with the Los Angeles classification^18^ was reported as follows: seven patients with Grade A (17.5%), six with Grade B (15%), one with Grade C (2.5%), and none in remaining patient collective. Hiatal hernias were specified with a median size of 2 cm, ranging from none to 6 cm. All patients, who received the RefluxStop^™^ device, were suffering from ineffective esophageal motility, according to the Chicago classification v4.0. With normal IRP values, patients presented with various combinations of in total seven failed (DCI < 100 mmHg) and weak (DCI 100–449 mmHg) contractions. The mean acid exposure time (AET) was 8.14 ± 2.53%, with a mean DeMeester score of 26.05 ± 16.17 (Table 2).

**Table 2:** Baseline characteristics.

Gender (%)	
Male	25 (62.5%)
Female	15 (37.5%)
Mean age (± SD)	48.93 ± 4.59
Mean years of GERD (± SD)	6.09 ± 1.47
Mean years of PPI Usage (± SD)	3.5 ± 0.84
Patients on PPI at time of surgery (%)	31 (77.5%)
Preoperative dysphagia	4 (10%)
Esophagitis	
Type A	7 (17.5%)
Type B	6 (15%)
Type C	1 (2.5%)
Type D	–
Median hiatal hernia size in cm (range)	2 (0–6)
Mean acid exposure time (± SD)	8.14 ± 2.53
Mean DeMeester score	26.05 ± 16.17

The preoperatively filled out GERD-HRQL questionnaires showed a mean heartburn score of 17.4 ± 2.07, a mean regurgitation score of 10.85 ± 2.85 and a total score of 32.83 ± 5.08. All patients were overall dissatisfied with current situation prior surgery.

### Serious adverse events

In this patient collective, one Clavian–Dindo 3b has to be reported: 68 year old female patient with a BMI of 32.1 kg/m^2^ underwent revisional surgery at the first postoperative day. In the routinely conducted barium swallow x-ray, the slipping of the newly reconstructed his angle and the RefluxStop device into the thoracic cavity was noticed. The patient presented with strong abdominal pain and had to be re-operated on the same day. During the laparoscopic revision, the stomach as well as the device were laparoscopically retrieved into the abdominal cavity, the device removed, the ripped cruroplasty re-sutured, and a Dor fundoplication performed.

There was one case of device migration noted in a 24 year old male patient, who lost the device via natural way 1 month after surgery. He experienced epigastric pain for 3 days and then was able to retrieve the device. The patient was asymptomatic and barium swallow CAT-scan showed no perforation. Surgery videos were analyzed, and possible explanation for this quick device migration might be a small hematoma at the device implantation site.

### Follow up

Patients were routinely checked up at three months with barium swallow x-ray. After first follow-up, already 87.5% (35) of all patients required no more PPI. Five patients are still taking PPI on a daily basis (p < 0.001), the need for daily PPIs decreased by 84%.

**Figure 1:**
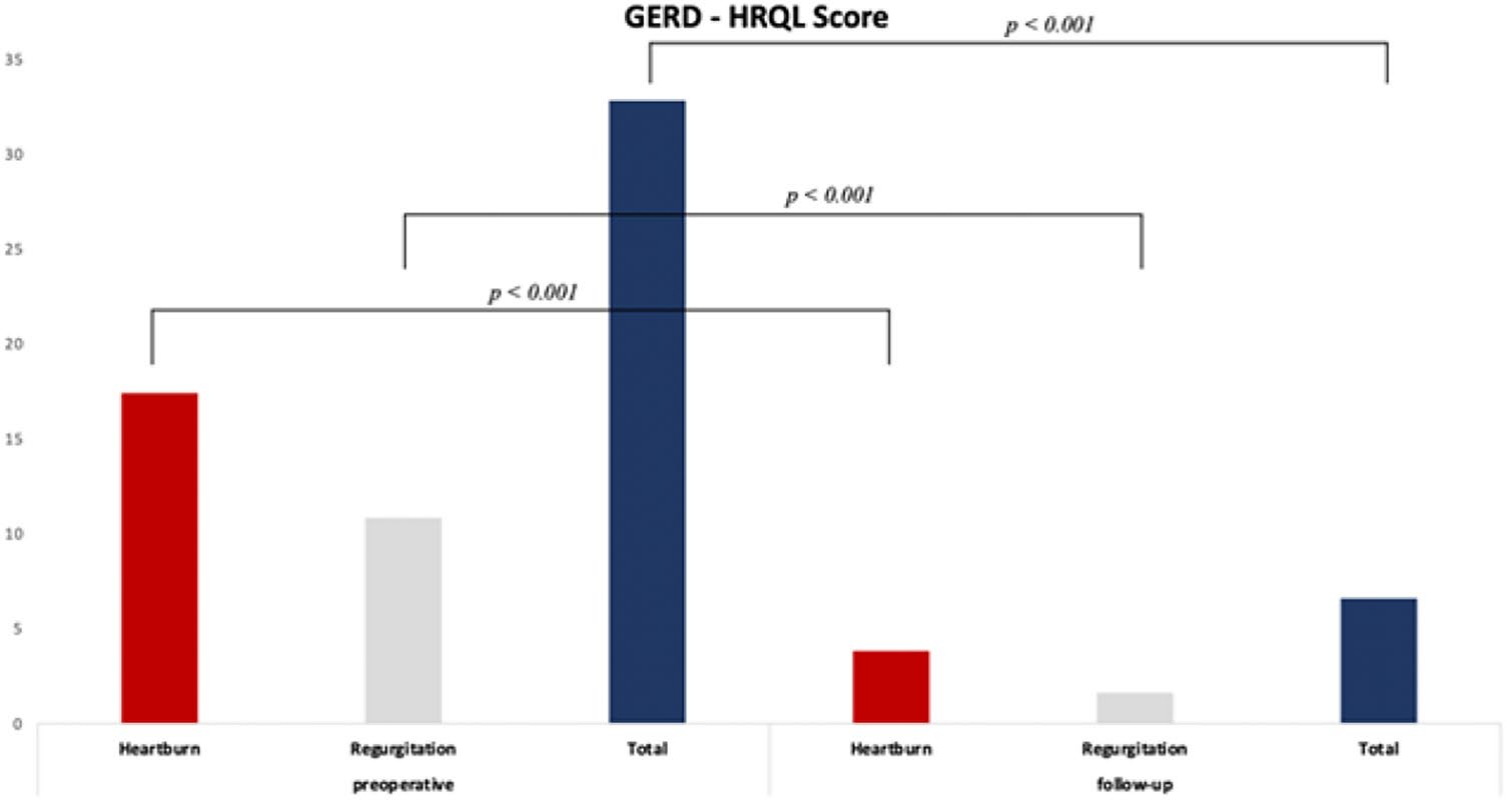
GERD-HRQL score pre- and postoperatively. Paired t-test was used for statistical analysis.

The heartburn score decreased from a mean of 17.4 ± 2.07to a mean of 3.83 ± 1.88 (p < 0.001) as well as the mean regurgitation score from 10.85 ± 2.85 to 1.63 ± 1.68 (p < 0.001). The GERD-HRQL total score amounts from 32.83 ± 5.08 to 6.6 ± 3.71 (p < 0.001) (Fig. 1).

**Figure 2:**
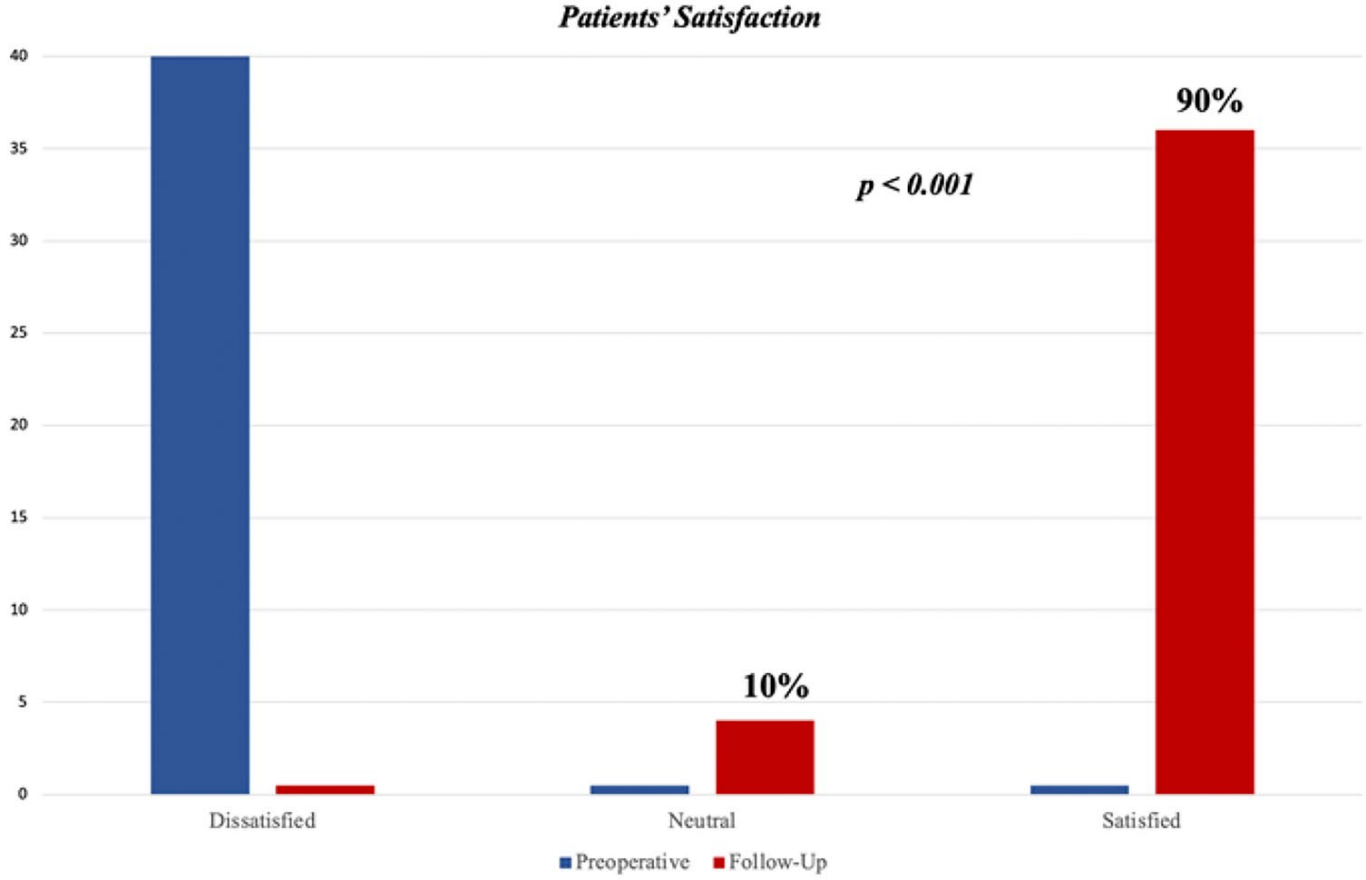
Patients’ satisfaction postoperatively.

The overall satisfaction increased: 36 patients (90%) felt satisfied after surgery, a dissatisfaction reduction of 89.19% was noted (Fig. 2 and Table 3).

**Table 3:** Postoperative outcome.

GERD-HRQL score (± SD)	Preoperative	Follow-up	
Heartburn score	17.4 ± 2.07	3.83 ± 1.88	p < 0.001*
Regurgitation score	10.85 ± 2.85	1.63 ± 1.68	p < 0.001*
Total (n = 40)	32.83 ± 5.08	6.6 ± 3.71	p < 0.001*
Dysphagia (%)	4 (10%)	1 (2.5%)	
Patients dissatisfied/neutral (n)	100% (40)	10% (4)	
Patients on PPI daily (%)	31 (77.5%)	5 (12.5%)	p < 0.001**

*Paired t-test.

**McNemar test.

## Discussion

With a prevalence rate of up to 33% and around 1 billion people suffering from GERD worldwide, whereas 30% of these are PPI therapy refractory, a necessity for symptom relief and optimal therapy is crucial^2^. For precise diagnosis, patients have to undergo functional testing with high-resolution manometry and multi-channel intraluminal 24 h impedance-pH monitoring^6,19^. If the patient has a pathological acid exposure time and/or a pathological amount of reflux episodes in 24 h, in accordance to the Lyon classification, GERD can be diagnosed^19^. GERD is caused by a various reasons, and goes hand in hand with the loss of normal anti-reflux barriers, such as the pressure of the LES, abdominal sphincter length, esophageal hiatus and His-angle^20^. Especially patients with IEM suffer from worse abnormal acid burden and have a higher probability to postoperative dysphagia due to diminished motility^5^.

85% of GERD patients have a shortened and structural defect LES as well as a shorter or depleted intraabdominal length, meaning that the LES moves into the thoracic cavity temporarily or permanently^21^.

The His angle has been a well discussed focal point in anti-reflux surgery, and there are many papers that debate its importance: Nissen, Lortat–Jacob and Collis, just to name a few, all made aware of the necessity for its repair and the resulting benefits^22–25^. As stated in the recently published new AFS hiatus grade classification, one of six main factors influencing the anti-reflux barrier is the acute angle of His and its role to preserving the flap valve^26^.

**Figure 3:**
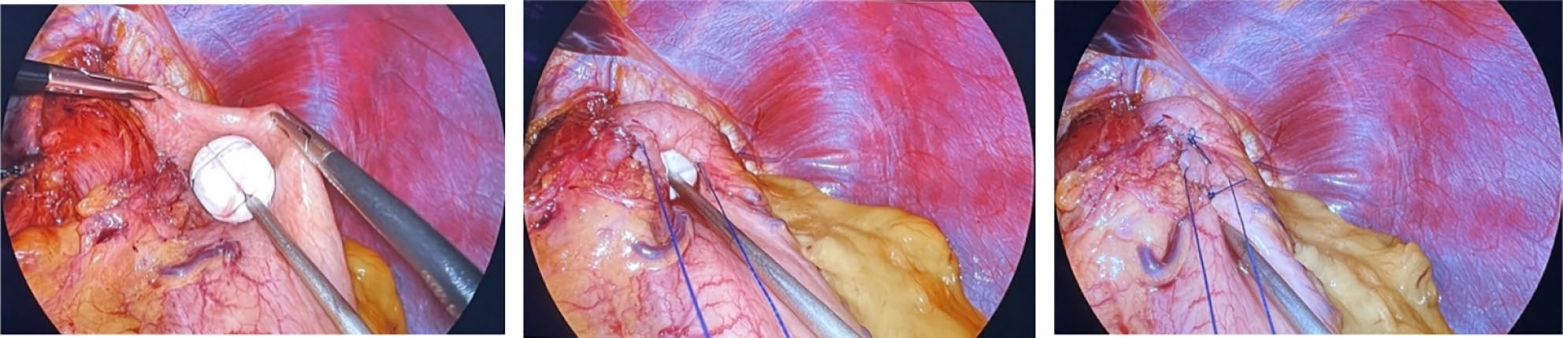
Intraoperative placement and fixation of the RefluxStop™ device.

The main goal of the Nissen fundoplication or LINX is to repair the internal sphincter by wrapping the fundus or the LINX device around the esophagus, thereby modifying the food passage pathway. This could potentially account for the occurrence of postoperative dysphagia. Especially patients suffering from an ineffective esophageal motility, which is defined by the Chicago classification v4.0, have shown to be more prone to postoperative dysphagia^6,27^. The RefluxStop^™^ device implantation combines the indispensable cruroplasty and the surgical repair of the His angle as two important GERD-inducing focal points whilst leaving the food passage way intact, while also inhibiting the movement of the LES into the thoracic cavity (Fig. 3)^15^.

Bjelovic et al. where able to show in this prospective, single arm multicentric clinical investigation in a cohort number of 50 patients promising results: patients undergoing this procedure, all suffering from long term GERD, had a significant decrease of acid exposure time in a 6 month follow-up, as well as improvement of GERD-HRQL scores, overall satisfaction, significant reduction of PPI usage postoperatively and most importantly, a dysphagia rate of 0%^15^. This rate was different in a 1 year follow-up study by Fringeli et al. where 15% of the operated patients required dilatation due to severe postoperative dysphagia^28^. We were able to show in our analysis of 40 consecutive patients, who underwent this procedure in two different institutions similar results in first follow-up: a significant reduction of PPI usage, significant decrease of GERD-HRQL score, minimal postoperative dysphagia rate, and an overall improvement of satisfaction.

All patients suffered from ineffective esophagus motility (IEM) to maximize the benefit of this complex subgroup of patients and to treat patients with an anti-reflux procedure without altering the food passage way. All patients underwent thorough examination via gastroscopy, functional testing as mentioned above, and GERD-HRQL questionnaire. The mean follow-up of patients was around 3 months, with clinical evaluation, barium swallow testing, and GERD-HRQL questionnaire.

Two serious adverse events were noted: one patient required revisional surgery on the first operative day, due to re-herniation of a part of the fundus and the RefluxStop device. Intraoperatively the hiatoplasty sutures seemed to be ripped open, the device was removed and a Dor-fundoplication as well as hiatoplasty was conducted. We believe that this complication occurred due to high BMI leading to higher intraabdominal pressure. This underscores the importance of precise patient selection for this novel procedure.

The other serious adverse event was a device migration one month after surgery: the surgical video was examined and as a possible reason for the migration might be a small hematoma, which occurred while creating the pouch for the device. The device migrated in one peace into the stomach, and left the body naturally. No further intervention was necessary.

There are several inherent limitations that need to be acknowledged due to the retrospective nature of the study. The absence of a control group in this study introduces a selection bias, as the findings are solely based on patients with IEM who underwent the RefluxStop procedure. Likelihood of complications, such as hiatal hernia recurrence, long-term effectiveness or implant migration, can be interpreted with some reservations, due to the short follow up period. Future studies are currently being conducted to compare patients with and without esophageal dysmotility as well as randomized controlled trials (RCTs) to compare RefluxStop with Nissen fundoplication. In this short follow—up analysis, the safety and efficacy of the innovative surgical technique have been demonstrated. However, randomized controlled trials (RCTs), long-term data as well as comparative data need to be conducted and reported, in order to further state its efficacy with the help of the GERD-HRQL as well as pH metry.

## Conclusion

In conclusion, the RefluxStop device is a promising new technology for the treatment of GERD. Given its minimally invasive nature and potential advantages for patients with esophageal motility disorders, initial findings suggest a low incidence of complications and quick recovery time during short-term follow-up. However, further studies and longer follow-up are necessary to prove long-lasting positive effects as a novel anti-reflux procedure.
